# Angle-to-angle and spur-to-spur distance analysis with high-resolution optical coherence tomography

**DOI:** 10.1186/s40662-020-00208-0

**Published:** 2020-08-16

**Authors:** Robert Montés-Micó, Pedro Tañá-Rivero, Salvador Aguilar-Córcoles, María Ruiz-Santos, María Dolores Rodríguez-Carrillo, Ramón Ruiz-Mesa

**Affiliations:** 1 Oftalvist, Alicante and Jerez de la Frontera, Alicante, Spain; 2 University of Valencia, Valencia, Spain

**Keywords:** Angle-to-angle, Spur-to-spur, White-to-white, Optical coherence tomography, Swept-source Fourier domain

## Abstract

**Background:**

To measure angle-to-angle (ATA) and spur-to-spur (STS) distances along six meridians using high-resolution swept-source optical coherence tomography (SS-OCT) and to compare those values with horizontal white-to-white (WTW) distance.

**Methods:**

68 eyes from 68 patients were quantitatively assessed with the Anterion SS-OCT (Heidelberg Engineering, Heidelberg, Germany). ATA and STS distances were measured with the SS-OCT’s B-Scan in six cross-sectional images corresponding to the vertical (6–12 o’clock), 1–7 o’clock, 2–8 o’clock, horizontal (3–9 o’clock), 4–10 o’clock and 5–11 o’clock meridians. WTW was measured horizontally with the device’s infrared camera. A Pearson correlation analysis was carried out to compare ATA and STS distances with WTW.

**Results:**

The largest values were found for the vertical meridian and the shortest for the 2–8 o’clock meridian, both for ATA and STS distances. No statistically significant differences were found between WTW, ATA and STS along the horizontal meridian (*p* > 0.1). However, ATA and STS showed statistically significant differences elsewhere, except for the horizontal and the 2–8 o’clock meridians (*p* > 0.05). Moreover, we found that ATA and STS varied significantly depending on the meridian being assessed, except for ATA at 4–10 versus 3–9 o’clock and for STS at 4–10 versus 3–9 o’clock and at 3–9 versus 2–8 o’clock (*p* > 0.1). R^2^ values ranged from 0.49 to 0.75 for ATA and STS at the different meridians, showing the best correlation at 3–9 o’clock meridian (0.64 and 0.75, respectively) and the worst at 6–12 o’clock meridian (R^2^ = 0.49 for both ATA and STS).

**Conclusions:**

ATA and STS distances vary radially, thus showing that the anterior chamber is vertically oval. Therefore, it is advisable to measure these two distances along the meridian to be used.

## Background

Measuring distances in the anterior segment of the eye has become important for clinical diagnosis but also to estimate the size of those phakic intraocular lenses (IOL) used to treat refractive errors. Implanting a lens with the proper size is extremely important to avoid undesired events such as decentration, rotation or inadequate vaulting [1, 2]. Phakic IOL size estimation traditionally relies on measuring horizontal white-to-white (WTW) distance, to which a constant value—usually between 0.5 and 1.0 mm—is added [3]. WTW can be easily measured using different methods such as surgical calipers, corneal topography, ocular biometry or, more recently, optical coherence tomography (OCT). However, the truth is that angle-to-angle (ATA) or spur-to-spur (STS) distances are the most appropriate values to calculate the size of anterior-segment phakic IOLs. Similarly, for posterior-segment phakic IOLs, their size should be calculated/estimated based on sulcus-to-sulcus distance. In this sense, internal distances are always preferable to external ones; over the past two decades several authors [4–26] have evaluated WTW, ATA, STS and sulcus-to-sulcus distances using different devices. Discrepancies between studies measuring horizontal WTW have been reported mainly due to manual or automatic measurements (being larger in this instance) [12]. Those differences may stem from the intrinsic difficulty to accurately define the location where the cornea ends and the sclera begins. In addition, previous studies have revealed that WTW distance shows a weak correlation with the sulcus-to-sulcus diameter measured with ultrasound biomicroscopy (UBM) [4, 5, 11, 13, 17–21]. Although UBM has facilitated the measurement of this distance, it is not very widespread in the clinical-practice setting, maybe because this technique is rather invasive and time-consuming. In contrast, the use of non-invasive high-resolution swept-source OCT (SS-OCT) has increased, despite the fact that sulcus-to-sulcus cannot be measured. Notwithstanding this fact, Oh et al. [11] reported that UBM-measured sulcus-to-sulcus and ATA diameters were significantly correlated for 4 meridians (45°, 90°, 135° and 180°). Other authors [14, 15, 17, 25] analyzing the horizontal meridian also support these findings, suggesting that angle diameter might be helpful for sulcus-size estimation. Some authors claim that it is better to estimate sulcus size using ATA rather than WTW as an alternative to measuring with an UBM [17]. Consequently, considering these outcomes, OCT-based ATA values may be useful and valid for posterior-chamber phakic lens size calculation.

High variability of WTW measurements (using manual calipers or automatic gray-scale steps devices) and their weak correlation with sulcus-to-sulcus distance support the idea of choosing other internal distances, such an ATA or STS, in order to minimize the above-mentioned variability and increase inter-parameter correlation. Moreover, measuring and comparing these distances along different meridians (due to the eye having a non-symmetrical shape) may help surgeons to accurately estimate the optimum IOL length and its placement, both for anterior- and posterior-segment lens models, thus reducing the likelihood of those adverse events that are secondary to improper sizing. To our knowledge, no studies have been published to date comparing these two distances (i.e., ATA versus STS) along different meridians measured with SS-OCT. For this reason, the purpose of the present study was to measure ATA and STS along 6 meridians using a high-resolution SS-OCT platform and to compare those values with horizontal WTW distance.

## Methods

A total of 68 eyes from 68 voluntary patients (21 males and 47 females) aged between 22 and 67 years were consecutively recruited for this study. This prospective study followed the tenets of the Helsinki Declaration and was approved by the Oftalvist Institutional Review Board (#2020–011). Exclusion criteria were having an ocular or systemic disease, poor fixation, a history of ocular surgery, or visual acuity below 20/25. The inclusion criteria were to be a phakic subject and being between 20 and 70 years old. Informed consent was obtained after they were given an explanation regarding the purpose of the study, and details on the measurement technique and on data handling and processing. A standard ophthalmological examination, including visual acuity and refraction, was performed before the measurements. Considering the reported similarities between a given person’s left and right eyes [27], only the subjects’ right eye was included in the study.

As for the measuring technique, a scanning high-resolution SS-OCT platform has been recently developed (Anterion, Heidelberg Engineering, Inc., Heidelberg, Germany). This instrument uses a 1300 nm (infrared) light source to obtain several B-Scans of the eye. It has an axial resolution < 10 μm, a lateral scan angle of up to 16.5 mm wide and a scan depth range of 14 ± 0.5 mm. The use of a long wavelength makes it possible to image the whole anterior segment and the lateral scanning SS-OCT allows for cross-sectional imaging providing data of different parameters analyzed. The instrument contains two imaging modalities: a lateral scanning SS-OCT and an infrared camera. The following parameters were evaluated with this instrument: WTW (defined as the horizontal distance between the nasal and temporal limbus, measured on the infrared-camera image), ATA (defined as the distance between two anterior-chamber angles in one B-scan, measured from angle recess point to angle recess point) and STS (defined as the distance between one scleral spur to the opposite scleral spur within one B-scan).

**Fig. 1:**
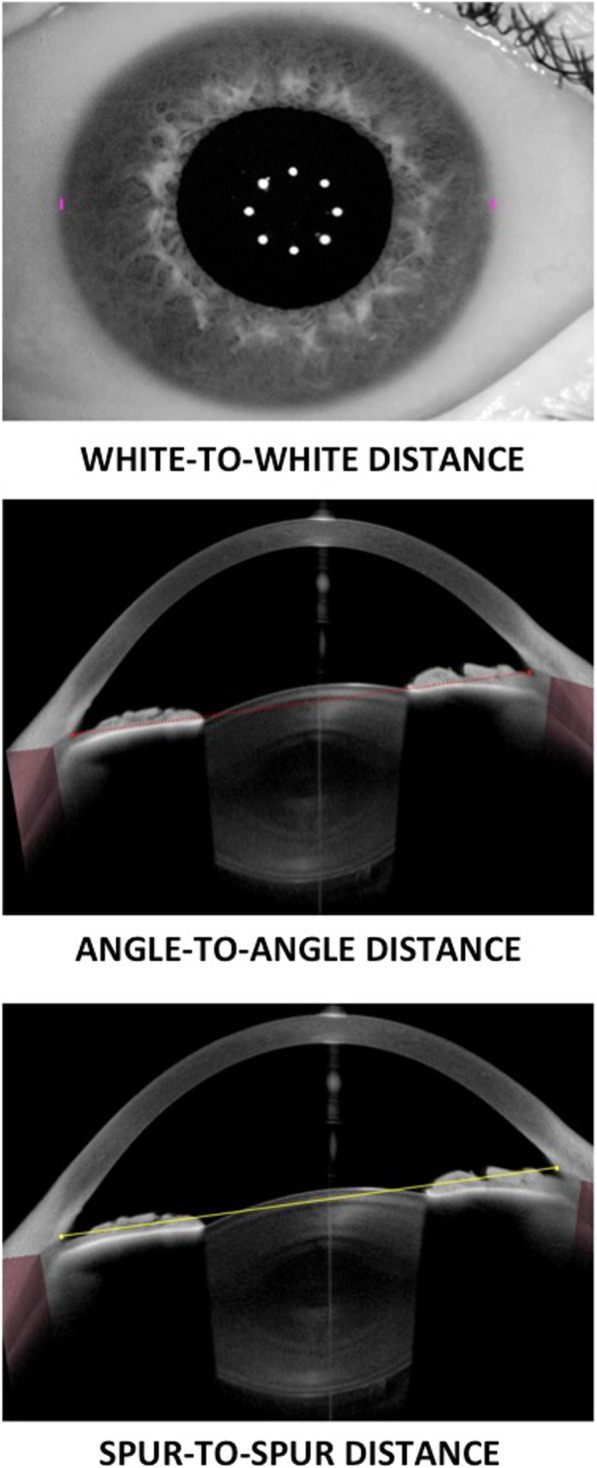
White-to-white, angle-to-angle and spur-to-spur distances at the horizontal meridian measured with the Anterion Swept-Source Optical Coherence Tomographer

A skilled operator took 5 consecutive measurements on each eye in the same session (mean values were used for the analysis). Each patient was positioned correctly on the chin rest, with their forehead leaning on the Anterion SS-OCT. Prior to each measurement, the instrument was calibrated according to the manufacturer’s recommendations. The following parameters were then measured: horizontal WTW (with the infrared camera) as well as ATA and STS, which were measured along 6 cross-sectional OCT B-scans: vertical (inferior-superior, 6–12 o’clock), 1–7 o’clock, 2–8 o’clock, horizontal (nasal-temporal, 3–9 o’clock), 4–10 o’clock and 5–11 o’clock. One trained observer was in charge of marking the scleral spurs in each image, which were defined as the inward protrusion of the sclera where a change in curvature of the corneoscleral junction was observed [28]. Figure 1 summarizes the different parameters (distances) that were analyzed in this study.

The statistical analysis was carried out using the SPSS software (version 22.0, IBM Corp., Armonk, New York, USA). All the measurements are given in the form of mean ± standard deviation (SD). Firstly, the ATA and STS values obtained for each meridian/scan were compared (i.e., the difference between these distances was computed); Secondly, we studied how ATA and STS varied within a given eye depending on the particular meridian/scan (variation of ATA or STS versus cross-sections). Before assessing differences, the normality distribution was checked by means of the Shapiro-Wilk test and the equal variance test by means of the Brown-Forsythe test. Statistically significant differences were detected with two-way repeated measures ANOVA test with Bonferroni post hoc analysis. *p* values below 0.05 were considered to be statistically significant. Differences between the three parameters (WTW, ATA and STS) were analyzed for the horizontal meridian. In addition, Pearson correlation analysis was performed to assess horizontal WTW distance versus ATA and STS distances at different meridians.

## Results

**Fig. 2:**
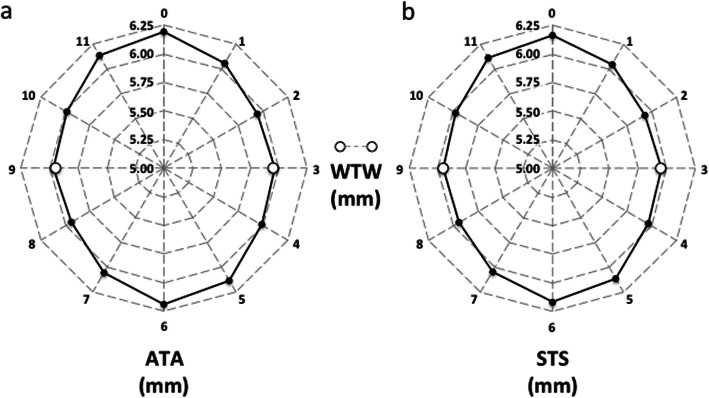
Angle-to-angle (**a**) and spur-to-spur (**b**) distances (black circles) measured at the different meridians. Horizontal white-to-white (WTW) distance (white circles) has been added to the radial distribution of both graphs for comparative purposes. Note that the radial axis starts at 5 mm

Sixty-eight participants (68 right eyes) were included in this study. Their mean age was 41.2 ± 11.2 years (ranging from 22 to 67 years). For all participating subjects, their measurement sessions were completed uneventfully. Table 1 summarizes the WTW values (only for the horizontal meridian), together with ATA and STS values (across 6 different meridians); they are all shown in the form of mean ± SD and range. Figure 2 shows a radial graphical representation of the values obtained for each meridian. From the diagram, the largest distances were found for the vertical meridian (about 12.3 mm) and the shortest for the 2–8 o’clock meridian (about 11.8 mm), both for ATA and STS measurements. The mean difference between these two meridians (largest-shortest) was 0.505 mm and 0.458 mm, for ATA and STS distances, respectively.

**Table 1:** Mean ± standard deviation (ranges) of the distances analyzed at different meridians

Parameter (mm)	3–9 meridian (horizontal)	6–12 meridian (vertical)	1–7 meridian	2–8 meridian	4–10 meridian	5–11 meridian
*WTW*	11.90 ± 0.38 (10.65 to 12.70)	–	–	–	–	–
*ATA*	11.93 ± 0.37 (11.05 to 12.94)	12.38 ± 0.38 (11.51 to 13.43)	12.11 ± 0.37 (11.26 to 13.10)	11.88 ± 0.37 (11.14 to 13.12)	11.97 ± 0.37 (11.20 to 12.86)	12.27 ± 0.38 (11.40 to 13.32)
*STS*	11.91 ± 0.35 (11.05 to 12.74)	12.33 ± 0.37 (11.39 to 13.11)	12.09 ± 0.36 (11.19 to 13.03)	11.87 ± 0.36 (11.08 to 12.86)	11.94 ± 0.36 (11.14 to 12.79)	12.23 ± 0.38 (11.33 to 13.18)
*Difference between ATA* vs. *STS (p value)*	0.016 (0.086)	0.053 (< 0.001^a^)	0.020 (0.031^a^)	0.006 (0.484)	0.024 (0.012^a^)	0.043 (< 0.001^a^)

*WTW =* white-to-white distance; *ATA* = angle-to-angle distance; *STS* = spur-to-spur distance

^a^statistically significant

**Fig. 3:**
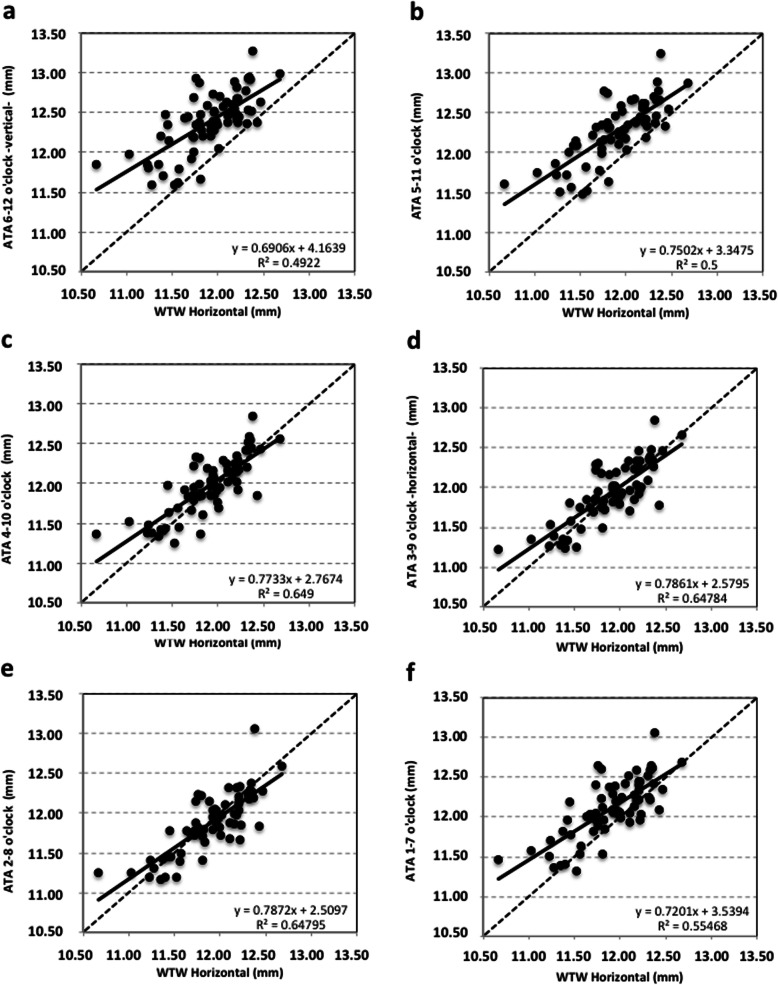
Scatter plot, equation, and Pearson correlation coefficient for horizontal white-to-white (WTW) distance versus angle-to-angle (ATA) distance measured at different meridians: vertical 6–12 o’clock (**a**), 5–11 o’clock (**b**), 4–10 o’clock (**c**), horizontal 3–9 o’clock (**d**), 2–8 o’clock (**e**) and 1–7 o’clock (**f**). Continuous line represents the best-linear fit and dotted line the equality

**Fig. 4:**
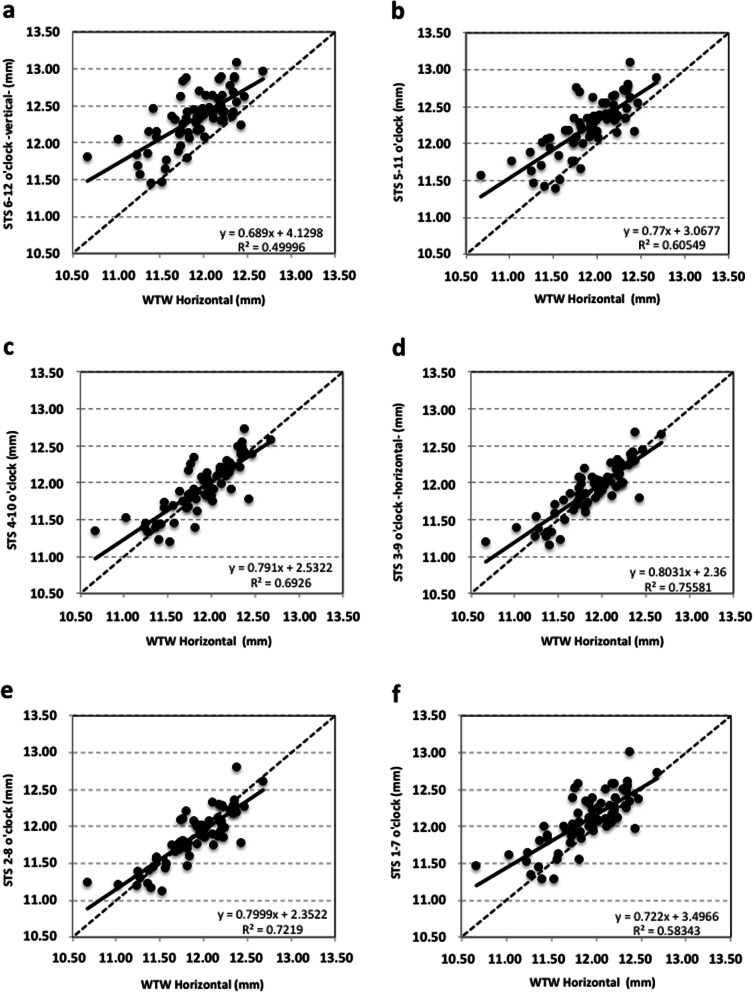
Scatter plot, equation, and Pearson correlation coefficient for horizontal white-to-white (WTW) distance versus spur-to-spur (STS) distance measured at different meridians: vertical 6–12 o’clock (**a**), 5–11 o’clock (**b**), 4–10 o’clock (**c**), horizontal 3–9 o’clock (**d**), 2–8 o’clock (**e**) and 1–7 o’clock (**f**). Continuous line represents the best-linear fit and dotted line the equality

No statistically significant differences were found between WTW, ATA and STS at the horizontal meridian (*p* > 0.1). Table 1 includes the differences between ATA and STS and the corresponding *p*-value. These differences turned out to be statistically significant for all meridians except for the horizontal and the 2–8-o’clock ones (*p* > 0.05). Table 2 shows the two-way ANOVA test with Bonferroni analysis to assess difference of means for ATA and STS as a function of the meridians compared. Statistically significant differences were found for all comparisons except for ATA at 4–10 versus 3–9-o’clock and for STS at 4–10 versus 3–9-o’clock and at 3–9 versus 2–8-o’clock (*p* > 0.1). For 31 eyes (45.6%), their WTW distance was larger than their corresponding ATA distance; for 3 eyes (4.4%), WTW and ATA values were equal, and for 34 eyes (50%), their WTW distance was shorter than the corresponding ATA. As for STS, these comparative outcomes were 33 (48.5%), 0 (0%) and 35 (51.5%) eyes, respectively. Figures 3 and 4 show the correlation between the horizontal WTW distance and the ATA and STS distances, respectively, obtained along different meridians, (R^2^ ranged from 0.49 to 0.75). The best correlation between distances was obtained for the 3–9 o’clock (horizontal) meridian for ATA and STS (R^2^ were 0.64 and 0.75, respectively). In contrast, the worst correlation was found for the 6–12 o’clock (vertical) meridian (R^2^ was 0.49 for both ATA and STS).

**Table 2:** Comparison between meridians for angle-to-angle (ATA) and spur-to-spur (STS) distances

Comparison between scans (hours o’clock)	Difference of means ATA (mm)	***P*** value	Difference of means STS (mm)	***P*** value
*6–12* vs. *2–8*	0.505	< 0.001*	0.458	< 0.001*
*6–12* vs. *3–9*	0.449	< 0.001*	0.412	< 0.001*
*6–12* vs. *4–10*	0.412	< 0.001*	0.383	< 0.001*
*6–12* vs. *1–7*	0.274	< 0.001*	0.240	< 0.001*
*6–12* vs. *5–11*	0.108	< 0.001*	0.097	< 0.001*
*5–11* vs. *2–8*	0.397	< 0.001*	0.360	< 0.001*
*5–11* vs. *3–9*	0.341	< 0.001*	0.314	< 0.001*
*5–11* vs. *4–10*	0.304	< 0.001*	0.285	< 0.001*
*5–11* vs. *1–7*	0.166	< 0.001*	0.142	< 0.001*
*1–7* vs. *2–8*	0.231	< 0.001*	0.217	< 0.001*
*1–7* vs. *3–9*	0.176	< 0.001*	0.171	< 0.001*
*1–7* vs. *4–10*	0.139	< 0.001*	0.142	< 0.001*
*4–10* vs. *2–8*	0.092	< 0.001*	0.075	< 0.001*
*4–10* vs. *3–9*	0.036	0.640	0.029	1.000
*3–9* vs. *2–8*	0.055	0.035*	0.045	0.178

*statistically significant

## Discussion

We believe this study is the first to measure and compare ATA and STS distances along 6 different meridians using a high-resolution SS-OCT platform. As mentioned in previous sections, there are several studies that measured WTW, ATA, STS and sulcus-to-sulcus distances using various devices, and then assessed the level of agreement across the different instruments and parameters; these studies’ main findings are summarized in Table 3 to facilitate their comparison.

**Table 3:** Mean values from previous studies obtained at different orientations where white-to-white (WTW) and angle-to-angle (ATA) or sulcus-to-sulcus or spur-to-spur (STS) were measured

Author	Eyes (n)	Device	WTW (mm)	ATA (mm)	Sulcus-to-Sulcus (mm)	STS (mm)
**Pop et al.** [4]	33/43	Caliper/50 MHz UBM^a^				
3–9 o’clock			11.87 ± 0.49	–	12.39 ± 0.58	–
**Werner et al.** [5]		Caliper				
6–12 o’clock	10^b^		10.95 ± 0.25	11.80 ± 0.40	11.60 ± 0.50	–
3–9 o’clock	12^b^		11.77 ± 0.40	11.77 ± 0.19	11.39 ± 0.32	–
**Rondeau et al.** [6]	28	50 MHz UBM				
3–9 o’clock			–	12.10 ± 0.31	12.35 ± 0.42	–
**Baikoff et al.** [7]	36	IOLMaster/OCT^c^	12.12 ± 0.44			
6–12 o’clock			–	12.40 ± 0.45	–	–
3–9 o’clock			–	12.10 ± 0.40	–	–
Oblique nasal			–	12.22 ± 0.39	–	–
Oblique temporal			–	12.23 ± 0.36	–	–
**Goldsmith et al.** [8]	40	Gauge/CAS OCT^d^				
3–9 o’clock			11.78 ± 0.57	12.53 ± 0.47	–	–
**Fea et al.** [9]	88	Orbscan/MRI				
3–9 o’clock			11.69 ± 0.40	–	11.70 ± 0.40	–
**Kohnen et al.** [10]	52	Orbscan/IOLMaster/ Visante OCT^e^				
3–9 o’clock			11.84 ± 0.41 (Orbscan IIz) 12.17 ± 0.45 (IOLMaster)	12.45 ± 0.53	–	–
**Oh et al.** [11]	28	Orbscan/ 35 MHz UBM^f^				
6–12 o’clock			–	12.00 ± 1.01	11.99 ± 0.73	–
3–9 o’clock			11.74 ± 0.42	11.55 ± 0.88	11.32 ± 0.72	–
Oblique nasal			–	11.80 ± 0.90	11.55 ± 0.77	–
Oblique temporal			–	11.75 ± 1.00	11.54 ± 0.75	–
**Piñero et al.** [12]	30	CSO/Visante OCT				
3–9 o’clock			12.25 ± 0.49	11.76 ± 0.52	–	–
**Kim et al.** [13]	20	Orbscan/ UBM 35 MHz^f^				
3–9 o’clock			11.78 ± 0.42	–	11.01 ± 0.68	–
**Reinstein et al.** [14]	40	Orbscan/ UBM 50 MHz^f^				
3–9 o’clock			12.06 ± 0.37	12.88 ± 0.42	12.85 ± 0.69	–
**Piñero et al.** [15]	20	Visante OCT/ UBM 50 MHz				
3–9 o’clock			–	12.23 ± 0.59 (OCT) 12.14 ± 0.52 (UBM)	11.92 ± 0.80 (UBM)	–
**Nemeth et al.** [16]	91	IOLMaster/ Visante OCT^e^				
3–9 o’clock			11.99 ± 0.47	11.43 ± 0.51	–	–
6–12 o’clock			–	10.72 ± 0.66	–	–
**Kawamorita et al.** [17]	31	Orbscan/ UBM 35 MHz^f^				
3–9 o’clock			11.65 ± 0.32	11.93 ± 0.44	12.06 ± 0.55	–
**Biermann et al.** [18]	16 (emmetropes) 21 (myopes)	Orbscan/IOLMaster/ UBM 50 MHz^f^				
6–12 o’clock	16 (emmetropes) 21 (myopes)		–	–	12.50 ± 0.33 12.52 ± 0.50	–
3–9 o’clock	16 (emmetropes) 21 (myopes)		11.83 ± 0.48/12.24 ± 0.57 11.66 ± 0.25/12.17 ± 0.26	–	12.15 ± 0.48 12.22 ± 0.48	–
Oblique 45°	16 (emmetropes) 13 (myopes)		–	–	12.20 ± 0.44 12.53 ± 0.34	–
Oblique 135°	16 (emmetropes) 13 (myopes)		–	–	12.21 ± 0.41 12.52 ± 0.34	–
**Gao et al.** [19]	Highly myopes	IOLMaster/ UBM 50 MHz^g^				
3–9 o’clock	38 (shallow AC) 35 (medium AC) 38 (deep AC)		11.46 ± 0.38 11.54 ± 0.31 11.68 ± 0.22	–	11.57 ± 0.32 11.77 ± 0.26 11.91 ± 0.23	–
6–12 o’clock	38 (shallow AC) 35 (medium AC) 38 (deep AC)		–	–	12.27 ± 0.48 12.52 ± 0.37 12.69 ± 0.36	–
**Petermeier et al.** [20]	50 (pseudophakic)	IOLMaster/ UBM 50 MHz^f^				
6–12 o’clock			–	11.65 ± 0.49	11.18 ± 0.57	–
3–9 o’clock			11.82 ± 0.35	11.43 ± 0.50	10.91 ± 0.53	–
Oblique nasal			–	11.49 ± 0.49	11.04 ± 0.57	–
Oblique temporal			–	11.47 ± 0.51	10.98 ± 0.58	–
**Reinstein et al.** [21]	50	Orbscan/ UBM 50 MHz^f^				
3–9 o’clock			11.83 ± 0.28	–	11.25 ± 0.50	–
**Erb-Eigner et al.** [22]	100	MRI				
3–9 o’clock			10.54 ± 0.76	10.43 ± 0.73	10.42 ± 0.76	–
**Hashemian et al.** [23]	273	Caliper/Orbscan/ UBM 35 MHz^g^				
3–9 o’clock			11.65 ± 0.37 (Caliper) 11.74 ± 0.42 (Orbscan)	–	12.13 ± 0.45	–
**Nakamura et al.** [24]	46	UBM 35 MHz/Casia2 SS-OCT^h^				
3–9 o’clock			11.72 ± 0.42	11.86 ± 0.50	11.78 ± 0.42	11.87 ± 0.43
**Ghoreishi et al.** [25]	58	Pentacam/ UBM 50 MHz^i^				
3–9 o’clock			11.87 ± 0.36	12.10 ± 0.33	11.87 ± 0.57	–
**Bruner et al.** [26]	65	Lenstar/Casia-1000 SS-OCT^j^				
3–9 o’clock			12.11 ± 0.40	–	–	11.87 ± 0.33
6–12 o’clock			–	–	–	12.11 ± 0.33
1–7 o’clock			–	–	–	12.07 ± 0.33
2–8 o’clock			–	–	–	11.91 ± 0.35
4–10 o’clock			–	–	–	11.87 ± 0.35
5–11 o’clock			–	–	–	11.98 ± 0.34
**Current study**	68	Anterior SS-OCT				
3–9 o’clock			11.90 ± 0.38	11.93 ± 0.37	–	11.91 ± 0.35
6–12 o’clock			–	12.38 ± 0.38	–	12.33 ± 0.37
1–7 o’clock			–	12.11 ± 0.37	–	12.09 ± 0.36
2–8 o’clock			–	11.88 ± 0.37	–	11.87 ± 0.36
4–10 o’clock			–	11.97 ± 0.37	–	11.94 ± 0.36
5–11 o’clock			–	12.27 ± 0.38	–	12.23 ± 0.38

*UBM* = ultrasound biomicroscopy; *OCT* = optical coherence tomographer; *MRI* = magnetic resonance imaging; *AC* = anterior chamber

^a^caliper for WTW and UBM for sulcus-to-sulcus measurements

^b^post-mortem eyes

^c^Holladay-Godwin gauge for WTW and OCT for ATA

^d^IOLMaster for WTW and OCT for ATA measurements

^e^Orbscan/IOLMAster for WTW and Visante OCT for ATA measurements

^f^Orbscan/IOLMaster for WTW and UBM for other measurements

^g^Caliper and Orbscan for WTW and UBM for sulcus-to-sulcus measurements

^h^UBM for sulcus-to-sulcus and Casia2 SS-OCT for other measurements

^i^Pentacam for WTW and UBM for ATA and sulcus-to-sulcus measurements

^j^Lenstar for WTW and Casia-1000 SS-OCT for sulcus-to-sulcus measurements

As for our study, we found no statistically significant differences between WTW, ATA and STS at the horizontal meridian (*P* > 0.1); in fact, the values were similar for the three distances (see Table 1). However, our mean values are different from those reported in previous studies (see Table 3 for detailed values). For instance, some of these studies concluded that the values differ and do not correlate [5, 8–14], while others [7, 16, 20] reported correlations between WTW and ATA. It is worth pointing out that a direct comparison between studies should be made with caution, considering the different methods used to measure these distances and the different eye samples (i.e., most studies included less than 50 eyes). It should also be noted that ours was the only study that measured all three parameters with the same instrument; the others made use of two or even three devices, which makes comparisons even more problematic.

The ATA vs. STS comparison for a given meridian yielded significant differences for all meridians except for the horizontal and the 2–8-o’clock ones (*p* > 0.05, see Table 1). For these two orientations, both distances can be considered to be equivalent. Nonetheless, the remaining orientations the differences ranged from 0.02 to 0.05 mm, which are clinically non-significant. Furthermore, for clinical purposes (i.e., lens sizing) we may consider both parameters (ATA and STS) to be similar for all meridians analyzed. Unfortunately, there are no studies in the literature comparing these two distances along different meridians; therefore, we could not compare our results with any previous data.

The analysis of how ATA and STS vary with meridian is graphically shown in Fig. 2. This figure shows how they change radially (specific values can be seen in Table 1) indicating that the largest values are found for the vertical meridian (about 12.3 mm) and the shortest for the 2–8-o’clock meridian (about 11.8 mm), both for ATA and STS distances. Changes were statistically significant for all meridians except for ATA at 4–10 versus 3–9 o’clock and for STS at 4–10 versus 3–9 o’clock and at 3–9 versus 2–8 o’clock (Table 2; *p* > 0.1). The difference between the largest and the shortest was 0.505 mm and 0.458 mm, for ATA and STA distances, respectively (*p* < 0.001). Table 2 shows mean differences for all comparison between meridians in ATA and STS distances. Our results agree with previous studies reporting data between vertical and horizontal meridians. For instance, Werner et al. [5] reported larger ATA values along the vertical meridian than along the horizontal one in post-mortem eyes, although the eyes evaluated were different for the vertical and horizontal meridians. Baikoff et al. [7] found that vertical ATA was greater than horizontal ATA by at least 100 μm in 74% of the eyes and by more than 300 μm in nearly 50% of the eyes. Oh et al. [11], using a 35 MHz UBM, reported statistically significant differences between vertical and horizontal ATA (*p* < 0.001), with a mean difference of 0.45 ± 0.40 mm. This corroborates with our study’s difference between vertical and horizontal ATA values; it was also 0.45 mm. Petermeier et al. [20], using a 50 MHz UBM, also found larger vertical ATA values than horizontal ones (i.e., 0.22 mm, with the differences being statistically significant). The exception to the rule was Nemeth et al.’s study [16], which reported that horizontal ATA was greater than the vertical ATA.

With respect to measuring STS, we have to consider, based on our results and those found by other authors, that the anterior chamber is vertically oval. Our STS outcomes and those found by Bruner et al. [26] also support this hypothesis. Both studies showed larger vertical than horizontal STS values: 12.11 versus 11.87 mm, and 13.33 versus 11.91 mm, for Bruner et al. [26] and us, respectively. Both studies analyzed a large sample (65 and 68 eyes, respectively) and used SS-OCT technology.

It is necessary to bear in mind that even though both ATA and STS varied radially, the horizontal values (ATA: 4–10 versus 3–9 o’clock and STS: 4–10 versus 3–9 o’clock and 3–9 versus 2–8 o’clock) did not change. This suggests that horizontal distances are more robust and unaffected by orientation than vertical ones, which is why any procedure requiring robustness to orientation should consider this meridian. However, in other scenarios, it is best to opt for the largest distance i.e., the vertical distance, which is about 0.5 mm larger. A difference of 0.5 mm between the vertical and horizontal distances is indeed important for some clinical decisions such as IOL size selection, keeping in mind that this size usually varies in steps of 0.5 mm. Biermann et al. [18] suggested that the axis of a posterior phakic IOL implantation should coincide with the sulcus-to-sulcus meridian measurement to avoid miscalculations, and that it is tempting to speculate that the postoperative risks would be reduced if the largest distance were used as the basis for IOL length calculation and if the lens were implanted vertically.

As mentioned in the *Introduction*, the sulcus-to-sulcus distance is the most appropriate parameter for posterior-chamber phakic IOL calculations. This distance can be measured directly only with UBM [4, 5, 11, 13–15, 17–21, 23–25] or MRI [9, 22], since OCT is unable to detect anterior-segment structures that are located behind the iris. However, in this study, as we have not used UBM or MRI, we are unable to compare our results with others.

Other studies have tried to correlate distances of anterior and posterior structures: two studies [14, 15] found a statistically significant correlation between WTW and sulcus-to-sulcus distance, whereas other reports found no or at most a weak correlation [4, 5, 9, 11, 13, 17–21]. These discrepancies may stem from the technique itself (the use of several images in UBM to be assembled to measure the entire sulcus) or from the specific sample (variables such as subject’s age, race, or refraction, among others). Notwithstanding the above, all studies agree that ATA correlates well with sulcus-to-sulcus distance for the horizontal [14, 15, 17, 24, 25] and also for other meridians [11]. Sulcus-to-sulcus distance also varies as a function of the selected meridian, with vertical-meridian distances being larger than horizontal ones [5, 11, 18–20]. These findings, with oblique cross-sections [11, 18, 20], also correlate with the ATA values reported by Bruner et al. [25] and with those yielded by the present study (see Table 3). A recent report by Nakamura et al. [24] used anterior segment parameters measured with SS-OCT for posterior phakic size determination showing excellent outcomes in postoperative vaults. Then, considering this, when UBM technology is not available, it might be better to estimate sulcus-to-sulcus using ATA than WTW distances. Finally, we want to point out that correlation does not mean equivalence, and this should be always kept in mind.

Surgeons often estimate STS by measuring horizontal WTW and then adding a constant value. This is based on the assumption that the anterior chamber is circular—instead of vertically oval—and that both distances are correlated. Different studies [4, 7, 20, 26] including ours, have shown that the anterior chamber is in fact vertically oval and that this assumption may affect the outcomes when the length of an anterior phakic lens is to be selected. Bruner et al. [26] recently analyzed this and concluded that adding 0.5 or 1.0 mmm to the horizontal WTW value in fact overestimates STS. The comparison needs to be done at different meridians because the footplates of the lens do not rest exactly at the axis of the lens insertion. Then, multiple comparisons should be carried out in order to properly assess differences between the horizontal and the other meridians. Figures 3 and 4 show the correlation between the horizontal WTW distance and ATA or STS distances, respectively, for different meridians. Note that there was a weak correlation for the different meridians except for the horizontal one where the regression line was similar to equality for both ATA and STS (Figs. 3 and 4, respectively). When we compare WTW with ATA or STS distances for the horizontal meridian, we found a similar percentage of eyes where ATA or STS was either larger or shorter than WTW (about 50%). Bruner et al. [26] reported that the vertical meridians tend to have less bias than the horizontal ones. They measured horizontal WTW with the Lenstar LS 900 optical biometer and STS with the CASIA SS-1000 OCT platform in 65 eyes (see Table 3). In our study, all measurements (horizontal WTW, ATA and STS at different diameters) were done using the same instrument, the Anterion SS-OCT platform. Our WTW value is smaller and similar to our horizontal STS, while Bruner et al.’s [26] is larger and closer to their vertical STS. As suggested above, the use of different instruments—especially for WTW measurements—may result in discrepancies. Moreover, the participants’ sex, age and racial differences may also play a significant role [29–32]. In our case, all patients were Caucasians (47 females) with a mean age of 41.2 ± 11.2 years, while in Bruner et al. [26] there were 29 Hispanic, 15 white, 12 black and 9 Asian eyes with a mean age of 43.14 ± 16.41 years (48 females). Taking this into account, we believe that more studies with larger samples and including different ethnicities should be carried out to properly understand the relationship between the distances that can be measured with different devices.

## Conclusions

In conclusion, we consider that it is advisable to have a direct measurement of the internal parts of the eye. If a SS-OCT is available, surgeons may consider direct measurements for ATA and STS at the meridian to be used. The use of different techniques results in significantly different distance values and, unfortunately, there are no studies comparing UBM with SS-OCT at different meridians. Future studies should be carried out to properly compare sulcus-to-sulcus with ATA and STS using both technologies for different axis. In addition, other SS-OCT devices should be also evaluated to analyze agreement between devices. These studies should be done with large samples with different ages and races. This may help to clarify controversies regarding the use of different measures for choosing the size and the placement of the phakic lens.

## Data Availability

All data generated or analyzed during this study are included in this published article.
